# TDF (Theoretical Domain Framework): how inclusive are TDF domains and constructs compared to other tools for assessing barriers to change?

**DOI:** 10.1186/1472-6963-14-S2-P81

**Published:** 2014-07-07

**Authors:** Hamideh Sarmast, Mirkaber Mosavianpour, Jean-Paul Collet, Niranjan Kissoon

**Affiliations:** 1 British Columbia Children’s Hospital (BCCH), Vancouver, British Columbia, Canada; 2 Child and Family Research Institute (CFRI), Vancouver, British Columbia, Canada; 3 Department of Pediatrics, University of British Columbia, Vancouver, British Columbia, Canada

## Background

Theoretical Domain Framework (TDF) provides an integrative conceptual model for assessing barriers to change. The TDF questionnaire has been applied by healthcare researchers in several countries for assessing barriers to performance improvement implying its possible usefulness in behavior modification.

## Aim

The purpose of this study is to review published literature on barriers to change using specific tools created without adhering to TDF theoretical framework and to investigate whether these tools incorporate domains and constructs of the TDF questionnaire.

## Materials and methods

We conducted a systematic literature review. We searched for papers in MEDLINE OVIDSP, PubMed, CINAHL, PsycINFO (that includes full text from PsycARTICLES), EBSCO Databases: Academic Search Complete and Google Scholar from beginning until April 2014. *Selection criteria* We included all papers that investigated barriers to change in health-related behavior or changing practice in health-related workplaces regardless of study design. Only papers published in English were included. We included in our review manuscripts that included the questionnaires either as attachment or in the content of the article. Duplicate studies were eliminated from the review by comparing authors’ names, type and location of study.

*Data abstraction* Study review and data abstraction were conducted by two reviewers working independently. Disagreements were resolved by discussion and referring unresolved issues to a third person.

## Results

Of 552 papers initially identified, 50 papers were selected for final review. The average number of items in the questionnaires assessing barriers was 19.2. In total the 50 questionnaires included 961 items, out of which 96.8% (930 out of 961) were covered by TDF. The “Environmental Context and Resources”, “Beliefs about consequences” and “Social Influence” were the domains of TDF that most frequently studied in the selected papers: 30.2%, 12.4% and 10.3%, respectively.

## Conclusions

This study confirms the validity of TDF framework to assess barriers to change: only 2.2% of 961 items identified were not covered by the TDF questionnaire. However, unclear boundaries between domains and the difficulty in identifying the appropriate construct were two issues identified that may be worth considering to improve the framework.

